# The application of data altruism in clinical research through empirical and legal analysis lenses

**DOI:** 10.3389/fmed.2023.1141685

**Published:** 2023-03-30

**Authors:** Teodora Lalova-Spinks, Janos Meszaros, Isabelle Huys

**Affiliations:** ^1^Clinical Pharmacology and Pharmacotherapy, Department of Pharmaceutical and Pharmacological Sciences, KU Leuven, Leuven, Belgium; ^2^Center for IT and IP Law (CiTiP), KU Leuven, Leuven, Belgium

**Keywords:** data altruism, clinical research, Data Governance Act, European Health Data Space, data protection

## Abstract

**Background:**

The legal framework for clinical research in the EU is complex and the lack of harmonization of the relevant legal and ethical rules remains one of the main challenges for stakeholders in the field. The recently adopted Data Governance Act (DGA) and the proposal for a European Health Data Space (EHDS) promise to solve the existing challenges with respect to access to and (re)use of personal data for research, but also risk to further complexify the field. The DGA introduced a novel mechanism – data altruism. Data altruism is understood as the voluntary sharing of personal and non-personal data, based on the consent of data subjects or the permission of natural and legal persons, without seeking a reward and for objectives of general interest. This study aimed to gain insights into the opinion of clinical research stakeholders on data altruism, and to critically discuss key issues pertaining to the application of data altruism from a legal point of view.

**Methods:**

Semi-structured interviews with (1) data protection officers (DPOs) and legal experts working with commercial and academic sponsors of clinical trials, (2) investigators, and (3) members of research ethics committees. Data underwent framework analysis. The legal discussion was comprised of legal doctrinal research with focus on the DGA, EHDS proposal, and the interplay with the EU General Data Protection Regulation (GDPR).

**Results:**

Fourteen experts took part in the interviews, more than half of which were DPOs/legal experts. Interviewees were based in seven EU Member states and the United Kingdom. The majority of participants were critical towards the data altruism mechanism and pointed out challenges and risks associated with its application.

**Conclusion:**

Although data altruism holds the potential to facilitate data sharing, its application in clinical research at the moment is still riddled with uncertainties. The interplay of the DGA rules with the provisions of the GDPR and the EHDS proposal are insufficiently clear and further efforts from the legislator are required to build a working, patient-centered, and research fostering data altruism system.

## 1. Introduction

The legal framework for clinical research in the European Union (EU) is complex (1, 2). The lack of harmonization of the relevant legal and ethical rules remains one of the main challenges for stakeholders in the field (3–5). Moreover, as clinical research heavily depends on the use and the reuse of personal data, the application of the European General Data Protection Regulation (GDPR) is of particular importance. Nevertheless, at present, the application of the GDPR scientific research regime is challenging and riddled with fragmentation (6–8).

The legal framework for clinical research is currently undergoing changes that bring both the promise to solve the existing challenges and the risk to further complexify the field. The changes are part of the measures that comprise the European Strategy for Data (9). The Strategy aims to create a single market for data through the establishment of common European data spaces in 10 strategic fields, including health. The aim is to ensure that more data becomes available for use in the economy and society, while keeping the companies and individuals who generate the data in control. In May 2022, the European Parliament and the Council adopted the Regulation on European data governance (Data Governance Act, hereafter DGA). The DGA aims to (1) facilitate the reuse of certain categories of data held by public sector bodies, (2) increase trust in data intermediation services and (3) foster data altruism across the EU.^1^ Specifically, the objective of the DGA is to set conditions for enhancing the establishment of the common European data spaces (10). One of the mechanisms foreseen in the DGA that has sparked particular attention by scholars, especially in the field of health research, is data altruism (11, 12).

As conceived by the DGA, data altruism is understood as the voluntary sharing of data based on the consent of data subjects to allow the processing of personal data pertaining to them, or the permission of data holders – both natural and legal persons^2^ – to allow the use of non-personal data, without seeking a reward and for objectives of general interest.^3^ Consents and permissions shall be collected and managed by data altruism organizations, registered and recognized in the EU.^4^ Such data altruism organizations will be able to collect relevant data directly from natural and legal persons, as well as to process data collected by others.^5^ They will be able to make the thus altruistically shared data available for use by data users (natural or legal persons)^6^, for objectives of general interest, including healthcare and scientific research. The concept of data altruism prior to the adoption of the DGA is discussed in Section 4.1 of this paper.

In the same month that the DGA was adopted, the European Commission published the proposal for a Regulation on the European Health Data Space (EHDS) (13), the first one of the envisaged common data spaces. The EHDS’ main goal is to increase control of natural persons over their electronic health data in healthcare, as well as for other purposes such as scientific research. Moreover, it aims to “ensure a legal framework consisting of trusted EU and Member State governance mechanisms and a secure processing environment” (14) that would allow researchers to access relevant electronic health data. The EHDS proposal builds upon existing relevant legislation, *inter alia*, the GDPR and the DGA. It establishes rules for the primary and secondary use of data. Whereas primary use (Chapter II) is focused on the governance of data uses in relation to the provision of healthcare and it elaborates rights and mechanisms complementing natural persons’ rights under the GDPR, secondary use rules (Chapter IV) are aimed at creating a permit-based system that allows health data to be shared for specific purposes, including scientific research. The provisions on secondary use in the EHDS proposal are also the first ones to include sector-specific rules about data altruism.

This article seeks to critically discuss the application of data altruism in clinical research and to put forward questions about uncertainties and gaps the recent (DGA) and proposed (EHDS proposal) laws trigger. To this end, the article addresses the new data altruism mechanism through empirical research (semi-structured interviews) and legal analysis lenses. First, we outline the materials and methods of our qualitative study. Afterwards, the results of the semi-structured interviews are reported. Finally, the empirical results are discussed, and are used as a starting point for in-depth legal analysis reflection on data altruism.

## 2. Materials and methods

### 2.1. Study design

Semi-structured interviews were performed in English *via* Microsoft Teams and Zoom between April and December 2021. The study was reviewed and approved by the Ethics Committee Research UZ/KU Leuven (S65106).

### 2.2. Research team and reflexivity

Reflexivity means sensitivity to the ways in which the researchers and the research process have shaped the collected data, including the role of prior assumptions and experience, which can have an influence on the study (15). The interview questions were designed by a researcher with a legal educational background (TL-S), who is currently working on a PhD project on the topic of clinical trials and data protection and has received additional training in empirical methods throughout her PhD trajectory. The questions were further discussed with a legal researcher with expertise on the relevant legal topics (JM, a postdoctoral researcher) and were discussed and critically revised by a professor in regulatory sciences with extensive expertise in qualitative methods (IH). TL-S and IH have a research interest in patient empowerment and patient engagement as well. All interviews were conducted by TL-S.

### 2.3. Participants and recruitment

Three stakeholder groups were targeted: (1) data protection officers (DPOs) and legal experts working with commercial and academic sponsors of clinical trials, (2) investigators, and (3) members of research ethics committees (ECs). The participants were recruited as part of a larger mixed-methods study which aimed to gain insights into the challenges experienced by relevant stakeholders in relation to data protection and obtaining (electronic) informed consent in clinical research. This study consisted of an online survey, disseminated widely across the EU and the United Kingdom, complemented by semi-structured interviews with survey participants who volunteered. The interview guide included questions on the DGA (then proposal), specifically on data altruism. Six experts with backgrounds in medicine, law, clinical research, and pharmaceutical sciences were involved in the pilot testing. Interviewees were presented with the definition of data altruism and asked to share their opinion on this new mechanism (Supplementary material 1). During the interview, more information about the mechanism (e.g., regarding the role of data altruism organizations) was provided to those participants who had not studied the DGA proposal in-depth, or were not aware of the new legislation.

The results of the larger study as regards data protection and (electronic) informed consent were published elsewhere (5, 16). The results as regards stakeholders’ views on data altruism are described in this article, as they provide an illustration of early reflections on the new mechanism and they constitute a suitable basis for further analysis on the legal implications of data altruism.

### 2.4. Analysis

Each interview was recorded and transcribed *ad verbatim* by a third party. The data were analyzed in accordance with the framework method (17), using the NVivo® software. The coding of all transcripts was performed by one researcher (TL-S), based on a working analytical framework. The same researcher – also responsible for conducting all interviews – had prior to that thoroughly familiarized with the discussions, *via* relistening to the audio recordings, rereading the transcripts, and comparing with their own reflective notes taken during each interview. In addition, legal knowledge was used to interpret and expand on the empirical findings. Key issues pertaining to data altruism and its interplay with other relevant rules, particularly contained in the GDPR and the EHDS proposal, were critically discussed through doctrinal legal lenses. The insights of clinical research stakeholders, presented in Section 3: Results, were used as a starting point for reflection on the legal issues (elaborated in Section 4: Discussion).

The quotes included in this paper were selected only from the part of each interview that discussed data altruism. Each quote is followed by a codename, consisting of a reference to the stakeholder group to which the interviewee belonged (see Table 1) and a number, e.g., DPO2.

**Table 1:** Demographic characteristics of the interviewed experts.

Stakeholder group	Code of the stakeholder group	Number of participants and %	Country where they were based	For EC only: national or local/regional	Active in international or national studies
DPOs/legal experts	DPO	*N* = 8 (57%)	Belgium, Czech Republic, France, Poland, United Kingdom	N/A	International: *n* = 6 National: *n* = 2
Investigators	INV	*N* = 2 (14%)	Germany, Portugal	N/A	International: *n* = 1 National: *n* = 1
Ethics committee (EC) members	EC	*N* = 4 (29%)	Belgium, Finland, Germany, Portugal	All were members of local/regional ECs	International: all
Total number of interviewees: *N* = 14					

## 3. Results

### 3.1. Demographics

Fourteen experts took part in the interviews. More than half were DPOs/legal experts (*n* = 8/14), followed by EC members (*n* = 4/14) and investigators (*n* = 2/14). The interviewees were based in seven EU Member states and the United Kingdom and were primarily active in international clinical studies (Table 1).

### 3.2. Views on data altruism

When presented with the definition of data altruism in the DGA proposal, most participants had a positive opinion about the idea of establishing such a mechanism but were critical toward the mechanism as actually conceived under the law. Their positive view toward the idea of data altruism was shown through the use of phrases such as: “I am very positive, it sounds like a potentially good development.” (DPO1), “I think it is an excellent idea.” (DPO3), “I think it is a good thing.” (DPO4), “I think it is a great idea.” (DPO8), “I think that for research it might be a great idea.” (EC3), “It is an opportunity.” (INV2). They expressed the view that the data altruism mechanism could potentially foster research in the EU by (1) facilitating the sharing of data and information, (2) motivating patients to be more willing to share their personal data, and (3) bringing more harmonization and resolving some of the legal uncertainty linked to the divergent national implementation of the GDPR in the field of scientific research.

Several interviewees were fully opposed to the idea of data altruism, as, according to them, it would not add anything new to the field. More specifically, they emphasized that similar mechanisms already exist, such as the French Health Data Hub.^7^ In the words of DPO2, the French Health Data Hub “works on the basis that if I go to the hospital, they register my data and send them to the Health Data Hub. The Health Data Hub then makes the data available to companies or researchers. (…) Instead of creating something new, why do we not improve what exists?” Participants also stressed that data altruism may further complexify the legal framework, particularly with the addition of a new intermediary (data altruism organizations), which may lead to data losses and time delays in research.

All respondents discussed at length the challenges and risks related to the data altruism mechanism. First, according to them, it would add more complexity with respect to three main topics: (1) the role and responsibility of the new data altruism organizations, (2) transparency requirements (e.g., would the third party (data user) need to inform the individuals that they have their data, or would it be enough that the data was forwarded by the data altruism organizations?), (3) consent and consent withdrawal (e.g., would data holders need to “return the data” (DPO1) if the consent provided to the data altruism organization was withdrawn?). As stated by DPO1, returning the data would be almost impossible in the life sciences sector “because we may rely on this data to get regulatory approval for a product.” According to several interviewees, the DGA proposal risks creating a “very bureaucratic system” (DPO1) in the life sciences field.

Second, participants opined that it is not so clear at this stage how a trusted environment for the sharing of data will be created. Data altruism organizations as gatekeepers were seen as “an excellent idea” (DPO3), but interviewees stressed the need for more safeguards. In addition, the national sensitivities toward data donation and trust in government authorities might be highly divergent, which would lead to unequal data altruism sharing across the EU.

Third, the interplay with the GDPR was criticized as not clear enough. As put by one DPO: “No matter how altruistic a patient is, maybe you are never going to be able to design an explicit and specific consent that will cover all secondary uses.”(DPO8) However, according to another DPO, the DGA creates an opportunity to implement the scientific research exception as a legal basis at EU level which would “bypass the need to be implemented in the national laws of 27 member states” (DPO5), and would thus bring further harmonization in the field.

Fourth, among our small group of ethics committee members, the view was prevalent that it should be carefully considered that data altruism might put too much responsibility on the patients who “do not always know what the consequences [of sharing their data] really are” (EC1).

Fifth, interviewees also found the implementation of data altruism potentially challenging from a scientific point of view. They believed that the datasets created through altruistic sharing would mostly be imbalanced, as certain types of individuals would be more willing to donate their data than others.

Finally, some interviewees considered the risk associated with unfair business models. For instance, an investigator brought up the concern that companies may start pooling datasets shared by data altruism organizations and selling them to other entities.

## 4. Discussion: Legal analysis

The empirical results showed that interviewed experts were mostly critical toward the data altruism mechanism. They pointed out challenges and risks associated with its application. Although many interviewees started by expressing a positive opinion, this opinion was linked to the idea of having a data altruism established through legal means *in abstracto* but was not dedicated to the mechanism in its current form (under the DGA proposal at the time of the interviews).

Below, key issues pertaining to data altruism and its interplay with other relevant rules, particularly contained in the GDPR and the EHDS proposal, are critically discussed through doctrinal legal lenses. The empirical research results offered the starting point for this reflection, and throughout the discussion, reference is made to them. However, the legal analysis goes further than the interviews, in order to provide a more in-depth analysis of the mechanism, as created under the adopted DGA.

### 4.1. The concept of “data altruism”

Several interviewees were opposed to the idea of establishing a data altruism mechanism with an EU legislative initiative, as according to them this would not add anything new to the field.

Although the concept of data altruism is indeed not novel, there is no common understanding of it in the wider political, legal and ethical literature. The related notion of data donation has long been part of the debate on personal data sharing for research purposes (including donating samples or tissue for science) (18). Interestingly, the DGA does not use the term “donation,” and the word may have been deliberately avoided by the legislator, as it implies ownership transfer, whereas the fundamental right to personal data protection cannot be contracted away (10, 19). Data altruism is also reminiscent of “data solidarity” (19), but the two notions are rooted in different understandings about how people act in the world. While altruism assumes that people act either selfishly, or in the interest of others, i.e., altruistically, data solidarity comes from the belief that acting in solidarity with others can empower both the giver and the receiver (20). Another similar term is “digital philanthropy,” which could be defined as the process of donating various types and forms of data by individuals and companies for the public good (21). In literature, the term “health-information altruists” was used to propose an approach in which individuals who have access to their health data can share them directly for research purposes with lower privacy guarantees (22).

The report prepared for the European Commission “Assessment of the EU Member States’ rules on health data in the light of the GDPR” highlighted only two existing national legal initiatives that were considered “some form of data altruism or data solidarity system”: the research-focused system in Denmark and the German Patient Data Protection Act, which provides insured persons with the option to make data stored in their electronic health record available for research (19). As shown above, data solidarity and data altruism are not the same concepts. The report also mentioned the French Health Data Hub – which was brought by some of the interviewed experts as an *ante litteram* example of data altruism - as one of the possible important players in the data altruism mechanism (19). Although the Hub could be perceived as an inspiration for data altruism organizations as conceived under the DGA, it is only one national example.

Based on the foregoing – and in opposition of the view held by most of the interviewees – the data altruism mechanism established with the DGA is in fact bringing a significant change to the existing legal framework. Going beyond the few and fragmented national approaches, the DGA aims to build a EU-level data altruism mechanism, underpinned by the aim to foster trust in data sharing. The introduction of a European data altruism consent form^8^ is a clear promise toward harmonization (23). However, the devil is in the details. Henceforth the next sections focus on the uncertainties and risks linked to the data altruism mechanism.

### 4.2. Data altruism consent

All participants agreed that data altruism would bring more complexity with respect to the topic of consent. In particular, the interplay with the GDPR rules was criticized as not clear enough.

In clinical research, consent is already a challenging issue. Two types of consent are relevant. First, informed consent to participate in clinical trials (under Article 28 of the Clinical Trials Regulation) – so-called research ethics consent – which follows from core ethical requirements of research projects involving humans as enshrined in the Helsinki Declaration. National provisions might require research ethics consent also for non-interventional studies. Second, consent as one of the possible legal bases under the GDPR for the processing of personal data.

As pointed out by the European Data Protection Board (EDPB), the two types of consent must not be confused (24), although in practice this is still often challenging for clinical research stakeholders (5). For instance, there is anecdotal and exploratory empirical evidence that ethics committees tend to advise clinical research stakeholders to rely on consent as the legal basis for the processing of data for research purposes (5, 25, 26), even though the EDPB has previously discouraged the use of this legal ground specifically in the clinical trials context due to the challenge to obtain a “freely given” consent in the meaning of the GDPR (24). Dove and Chen coined the term “consent misconception” to describe the scenario whereby, because research ethics consent is the favored mechanism and the key ethico-legal norm in research ethics governance, researchers perceive that this must also be the case for data protection purposes (27). However, a favored position for GDPR consent in the health research context contradicts both the GDPR (where such a privilege is not established), and the reality of research itself where for scientific, methodological, ethical and legal reasons researchers may prefer to rely on one of the other available legal bases.

Data altruism consent has been recognized by some scholars as a “new model of consent,” e.g., (12), although the DGA claims it falls “within the meaning of” the relevant GDPR provisions (Article 6(1) (a), Article 9(2) (a)) and it should be in compliance with the requirements for lawful consent (Articles 7 and 9 GDPR).^9^ Based on a reading of the relevant recitals and articles of the DGA,^10^ the new regulation does not intend to establish a new *type* of consent, not in the way that research ethics consent and consent under the GDPR are two distinct concepts. Nevertheless, the interplay between the DGA and the GDPR is indeed unclear at present, as the interviewees brought up. In the literature, questions have already been raised about the relationship between data altruism consent and the existing consent requirements under the GDPR (12, 28).

First, although the DGA reiterates the opening for broad consent for scientific research, offered by recital 33 GDPR,^11^ it does not provide the much-needed further guidance on the usability of broad consent.^12^ Moreover, the adopted version of the DGA did not remedy a weakness of the GDPR noted by the EDPB and the EDPS in their Joint Opinion 03/2021, namely that the specification of broad consent is not part of the substantive part of the regulation, and that it shall be accompanied by a clear distinction between (1) consent to areas of scientific research, (2) further processing for scientific or historical, or statistical purposes, and (3) the processing for the purposes of general interest (29).

Second, the DGA complicates the understanding of the notions of “purpose” and “processing operation.” Pursuant to the GDPR, consent is attached to one or more specific purposes.^13^ However, Article 25(3) DGA provides that “data subjects are able to give consent and withdraw consent from a *specific data processing operation* (…)” [authors’ emphasis]. Purpose and processing operation are not equivalent, as several processing operations can be performed for one purpose, and vice versa. For instance, in a clinical trial, one processing operation can be performed to answer a research question and to comply with a legal obligation (26). The authors of the CiTiP White Paper on the Data Governance Act discovered, in this regard, three possible interpretations of the DGA aim (11). Namely, they asked (1) whether the DGA endorses granular consents within one project, for which the data subject initially has provided a broad consent, (2) whether the DGA affords data subjects the possibility to give broad consents for a series of specific projects that serve one purpose, or (3) whether the DGA endorses the new “step-based approach” of the Court of Justice of the European Union (CJEU) in its recent case law on joint controllership, in which a processing operation-based understanding of consent appeared to be supported (30). All of these questions were formulated when the DGA was still a proposal (June 2021), but the final version of the adopted regulation did not provide an answer.

Third, historically, much emphasis has been placed on consent as a mechanism for individual empowerment. Nevertheless, the appropriateness of consent as a legal basis has been questioned in the case of clinical research. EDPB and the European Commission discouraged reliance on it for the processing of personal data in clinical trials due to the challenge to satisfy the criterion “freely given” in the meaning of the GDPR (24, 31). As discussed by the EDPB, depending on the circumstances of the clinical trial, situations of power imbalance may occur between sponsor/investigator (as data controller) and participants (data subjects) (24). For this reason, data controllers in clinical research increasingly rely on other legal bases, such as legitimate interests of the controller^14^ and public interest^15^. Moreover, the EHDS proposal also puts a strong emphasis on moving away from consent as an empowering mechanism, specifically when it comes to secondary use of data.^16^ Pursuant to Recital 37 EHDS proposal, the EHDS provides the legal basis in accordance with Articles 9(2) (g), (h), (i), and (j) GDPR for the secondary use of health data.

Related to the foregoing, the interplay between data altruism consent and consent as a requirement for participation in health research (understood broadly, going beyond interventional clinical trials) remains unclear. The data altruism consent should neither replace nor do away with the consent to participate in medical research projects, which means that research ethics consent shall additionally have to be collected in any context that implies participation in non-interventional retrospective medical research projects and when this is foreseen by national law. No guidance has been provided so far on how obtaining research ethics consent to conduct research on altruistically shared data will be organized in practice. For instance, data users to whom data altruism organizations would make data available (in anonymized or at minimum, pseudonymized format), would not be able to contact each individual altruistic data subject to obtain research ethics consent. Data users might be able to rely on Principle 32 of the Helsinki Declaration, which offers an exemption from obtaining research ethics consent in exceptional situations (i.e., where consent would be impossible or impracticable to obtain), provided that a research ethics committee has approved the research study (32). However, the Helsinki Declaration is not a legally binding instrument, and its principles have not been codified equally in the national laws of EU Member States. It is to be expected that data altruism organizations themselves would be responsible for obtaining research ethics consent together with the data altruism consent. Additionally, the role of ethics committees in the evaluation of projects relying on or incorporating altruistically shared data, has so far also not been subject to in-depth scholarly or policy debates.

Finally, the application of data altruism consent in the context of further processing of personal data is also not made sufficiently clear in the DGA. Recital 50 of the DGA specifies that “*typically*, data altruism would rely on consent of data subjects (…)” [authors’ emphasis]. A textual interpretation of the provision, and specifically of the use of the word “typically” in it, would be of interest here. Merriam-Webster provides two definitions for “typically”: (1) “on a typical occasion: in typical circumstances,” and (2) “in a typical manner.”^17^ Synonyms of “typically” include “commonly,” “generally,” “normally,” “usually” and “ordinarily.”^18^ By considering “typically” as denoting the frequency of data altruism relying on consent, two conclusions could be made.

On the one hand, the recital might suggest that data altruism would be “usually” focused on the re-use of personal data (rather than non-personal data). Such a conclusion appears to be supported by the European Commission’s preparatory work (9, 33) and has been suggested by the authors of the CiTiP White paper on the Data Governance Act (11).

On the other hand, the use of “typically” might also mean that consent, although the preferred legal basis for the processing of personal data for altruistic purposes, is not the only possible legal basis – i.e. while data altruism might “usually” rely on consent, it could also rely on other legal bases. Although such a conclusion appears confusing in the scope of primary use of personal data (i.e., it begs the question of how one can be altruistic if they did not voluntarily provide their personal data to the data altruism organization in the first place), a possible explanation can be found when considering the case of further processing of personal data. Recital 50 DGA touches upon further processing and highlights the presumption of compatibility in particular: “Article 5(1), point (b), of Regulation (EU) 2016/679 specifies that further processing for scientific or historical research purposes or statistical purposes should, in accordance with Article 89(1) of Regulation (EU) 2016/679, not be considered to be incompatible with the initial purposes.” However, Chapter IV of the DGA does not provide any more guidance about further processing in the scope of data altruism activities. Related to the foregoing, two main questions come to mind.

*(1) How to delineate primary and secondary use of personal data in the context of data altruism?*

A strict interpretation of the GDPR rules would mean that the mere collection of personal data by data altruism organizations, could be considered primary use,^19^ while any subsequent processing operation performed with the data constitutes secondary use or further processing. Following from this, making personal data available to data users would also be classified as further processing (secondary use) and as such would have to be in compliance with the relevant GDPR provisions.^20^ For instance, the data altruism organizations would have to conduct the compatibility test foreseen in Article 6(4) GDPR prior to transferring the data to the data user.

However, data altruism consent, as foreseen in recital 50 DGA and Article 25 DGA, appears to encompass the processing of personal data also by data users, once the data has been shared with them (notably because data subjects can withdraw their consent from “specific processing operations,” as discussed above). Therefore, an alternative interpretation of the DGA provisions could be that processing operations by data users (who receive data from data altruism organizations) would also constitute primary use. If that would be the case, then the GDPR rules for further processing of data would not apply. To show how this interpretation is possible, we use the example in the field of biobanks, provided by Becker et al. in a recent paper that comprehensively analyzed the secondary use of data through the lens of the GDPR. Population biobanks often collect and store personal data alongside human and tissue samples, with the aim to enable external entities to use these data for, e.g., research. The aim of population biobanks thus closely resembles the goal of data altruism organizations. Becker et al. argued that in the case of such biobanks, the transfer of personal data to biomedical researchers would not constitute further processing because “the intention to make the data available was the primary purpose driving the collection” (34). At the same time using the data by the third party would also constitute primary use. As no further processing would be taking place, none of the parties involved (biobanks and biomedical researchers) would need to comply with the GDPR rules for further processing of personal data (34).

Finding an answer to the question of how to delineate primary and secondary use of data in the context of data altruism is crucial for legal certainty, as the two possible interpretations of the DGA rules lead to two extreme understandings of how the GDPR should be applied by data altruism organizations – either the rules on further processing would need to be complied with, or not.

*(2) Which would be the valid legal basis for the processing of personal data by data users once they have received data by data altruism organizations?*

The answer to this question would depend on finding a solution to the question above. If the processing conducted by data users were considered primary use, then the valid legal basis would be the data altruism consent. However, if the processing conducted by data users would be considered further processing (secondary use), it is not clear how much freedom they would have in the choice of a legal basis. At present, one of the most contested questions in literature and practice is whether a new legal basis should be chosen for secondary use, or whether the processing could continue to be based on the legal ground used for primary use (if specific conditions apply).^21^ In the case of scientific research, Recital 50 DGA suggests that the presumption of compatibility can be relied upon, and moreover, the use of the word “typically” at the beginning of the recital, implies that consent would not be needed. However, the application of the presumption is currently riddled with uncertainties (5), and further authoritative guidance from the EDPB is still pending (24).

### 4.3. Data altruism with personal and non-personal data

The relevant provisions of the DGA make a clear distinction between data altruism with personal and non-personal data. Personal data can be made available by data subjects only based on consent, whereas non-personal data can be made available by data holders (both legal entities and natural persons) on the basis of permission.^22^ Under the DGA rules, legal entities can share only non-personal data altruistically.^23^

Technological advancements and the high threshold for data to be considered anonymized make the effectiveness of anonymization questionable (35). Data that are at present considered anonymous (and thus non-personal) might become identifiable in the future, and this is particularly the case in the field of rare diseases research. Therefore, legal entities who were altruistic with non-personal data they held could become incompliant with the applicable data protection rules, if in the future the data turns out to be identifiable and if there was no valid legal ground for the processing (sharing) of such data under the GDPR. It is thus unclear what the incentive for clinical trials sponsors would be to participate in data altruism activities. Moreover, whereas data altruism consent is aimed to be harmonized *via* the European data altruism consent form, a similar harmonizing effort is not envisaged for permissions, potentially introducing a risk for a fragmented legal framework.

### 4.4. Operationalizing data altruism

For data subjects to be altruistic with their personal data, they should first hold such data. According to the European Commission, one of the main goals of the European Strategy for Data is to provide that every citizen can “ensure the portability of his or her data” (9). Moreover, the DGA introduces a specific category of data intermediation services that offer their services to data subjects and would assist them in exercising their rights under the GDPR, *inter alia* and specifically named, data portability.^24^ In the cases where health data would be first collected and processed by another entity (e.g., a hospital, an academic or commercial sponsor of a clinical trial), the right to data portability could theoretically be the way through which data subjects would be able to hold their personal data and to act altruistically.

Under the GDPR, the right to data portability, namely the right to receive one’s data and to have it transmitted to another data controller, is afforded only in limited cases. Data should have been initially provided by the data subject (i.e., inferred data is excluded from the scope of the right), it should have been processed on the legal bases of consent or performance of a contract, and the processing must be carried out by automated means (i.e., excluding paper files).^25^ However, in clinical research, other combinations of legal bases have been reported to be more preferred (such as legal obligation, public interest, or legitimate interest), depending on the case at hand or the specific national legal framework (19, 24). In addition, in cross-border clinical trials, sometimes different legal bases legitimize the processing of personal data in the different countries where investigational sites are open. Using different legal bases in the scope of one cross-border clinical trial potentially creates inequalities between patients from different countries, as different legal bases come with diverse application of the data subjects’ rights (5, 26). Thus, it could be the case that within the same clinical trial, some patients could exercise the right to data portability (and, linked to this, could provide data altruism consent for the reuse of their data), whereas others would not have this possibility.

If the use of data portability is so limited (36), this begs the question how data altruism can be practically executed in clinical research.

Two new legislative proposals, also part of the European Strategy for Data measures, appear to provide a solution. In particular, the EHDS proposal and the Data Act (DA) proposal (37). Both acts promise to broaden the scope of the right to data portability in varying degrees.

On the one hand, the EHDS proposal strengthens the right to data portability by opening it to inferred and derived data (e.g., medical examinations), and by establishing that it applies irrespective of the legal basis for the original processing of the data.^26^ In addition, the scope of portability under EHDS encompasses both personal and non-personal data. The DA proposal, on the other hand, broadens the right to data portability in the context of Internet-of-Things products by affording it to both natural *and* legal persons; it also makes it apply to data processed on any legal basis; and finally, it includes both actively provided as well as passively observed personal data in its scope^27^ (see also Table 2 for a detailed overview).

**Table 2:** The three versions of the data portability right, as established in the GDPR (Article 20), the Data Act proposal (Article 4), and the EHDS proposal (Article 3(8)).

Applies to		GDPR	Data Act proposal	EHDS proposal
Personal data	*- Provided by the concerned data subject*	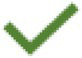	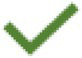	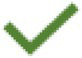
	*- Inferred data*	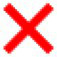	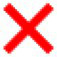 ^1^	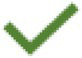 ^2^
Non-personal data		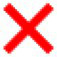	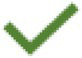	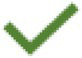 ^3^
Irrespective of the legal basis for the processing of personal data		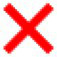 ^4^	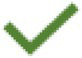	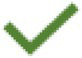
For care and research, primary and secondary use		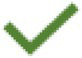	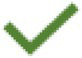	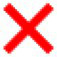 ^5^
Legal persons (in addition to natural persons)		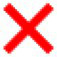	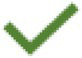	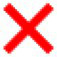

^1^Although the Data Act proposal excludes “inferred” and “derived” data from its scope of application (Recital 14 DA proposal), it nevertheless seems to go beyond the scope of the GDPR, as it applies to both “actively provided” and “passively observed” data (Recital 31 DA proposal). However, the concepts “actively provided” and “passively observed” are not defined in the DA.

^2^EHDS includes “inferred” and “derived” data, as well as “observed” data and recorded data by automatic means (Recital 5 EHDS proposal).

^3^EHDS introduces the notion of “electronic health data” (EHD) which encompasses both personal and non-personal (electronic health) data (Article 2(2)(a-c) EHDS proposal). However, it is not sufficiently clear whether data portability applies to both personal and non-personal EHD (which is suggested by Art. 3(8) EHDS Proposal), or only to personal EHD as explicitly outlined in Recital 11: “Natural persons should be further empowered to exchange and to provide access to *personal electronic health data* to the health professionals of their choice, going beyond the right to data portability as established in Article 20 of Regulation (EU) 2016/679” [authors’ emphasis].

^4^Under the GDPR, portability is limited only to data processed based on consent or contract.

^5^It appears that data portability under the EHDS is limited to primary use of data in the health and social security sector, see Article 3(8) EHDS proposal: “Natural persons shall have the right to give access to or request *a data holder from the health or social security sector* to transmit their electronic health data *to a data recipient of their choice from the health or social security sector*, immediately, free of charge and without hindrance from the data holder or from the manufacturers of the systems used by that holder” [authors’ emphasis].

At first glance, it appears that by relying on the provisions of either the EHDS proposal, or the DA proposal, participants in clinical research would be able to be altruistic with their personal (and even non-personal) data. However, it remains to be seen how the three versions of the right to data portability would apply in practice, and a careful examination of the new provisions is highly necessary in order to assess whether they truly provide individual patient empowerment. For instance, scholars have already voiced concerns regarding the opening of the right to data portability to legal entities under the Data Act proposal (38). Moreover, the data portability enshrined under the EHDS proposal appears to be limited only to the healthcare setting^28^, thus excluding portability of personal data for research purposes.

Although not promoted in policy documents, there is another possible tool for the operationalization of data altruism that is not limited in the ways that portability is, namely the right of access.^29^ The right of access consists of the right of the data subject to obtain confirmation from the controller regarding whether or not personal data concerning him is being processed, and, if that is the case, to obtain a copy of the personal data undergoing processing.^30^ By obtaining a copy of their own data, data subjects would be in a position to make it available to data altruism organizations. However, there could be various legal, statutory and professional limitations, or ethical barriers that could potentially impede the sharing of the full scope of available information with the data subject. Genetic research provides for a good practical example, as European ethical standards, for instance, mandate that genetic test results may not be provided to patients without proper counseling (39). Additionally, for professional reasons, sequence data are not part of the medical record and providing such information may expose the controller to civil and/or criminal liability (40).

It is not the aim of this paper to evaluate in detail the applicability of the right to data portability and the right of access as tools that enable data altruism. However, our contribution already highlights the barriers and uncertainties that await data altruism in (clinical) research, in the absence of more concrete measures for its implementation.

Finally, the hurdles for operationalizing data altruism in clinical research could also be hinting at a particular policy choice, i.e., that data altruism was not intended to be used in clinical research specifically, even if there is a clear objective to have the mechanism employed in health research in general (as the EHDS proposal – a *lex specialis* – provides further rules on altruism).

Until the present moment, no policy documents have provided a clear indication of such a choice but considering the specificities of clinical trials conduct – not only from a legal point of view, but also from a practical and organizational point of view – it is valuable to entertain such a conclusion. In particular, fully operationalizing data altruism – be it through a broadened data portability right or another tool – could present the same risks to the final data analysis of a clinical trial, as currently exist in relation to GDPR consent withdrawal.

As discussed by representatives of the European Organization for Research and Treatment of Cancer (EORTC), an academic sponsor of clinical trials, in case consent is used as the valid legal basis for the processing of personal data in a clinical trial, and some study participants withdraw their consent at a later stage of data collection, it is not clear whether their data can still be included in the final analysis. Similarly, it is also not clear whether the results of the interim analysis can be re-done to verify its correctness, or whether the dataset (with the data of people who withdrew) can be used in the scope of an inspection or an audit of the competent authorities (26). Consent withdrawal and returning the data in the life sciences sector was brought as an important concern also by the experts interviewed for this study. It could be argued that having the unlimited opportunity to move one’s personal data from the clinical trial (in which one is participating) to a data altruism organization would create uncertainties in relation to the use of the clinical trial dataset.

However, the objectives of the European Strategy for Data – and of the DGA and EHDS proposal in particular – are two - fold: namely, facilitating access to and reuse of data for, *inter alia*, research, while empowering individuals with respect to control over their personal data. A data altruism mechanism which is fully open to individuals in some contexts, but limited for use in others (such as, presumably, clinical research), would appear to not be completely aligned with the empowering aim of the new legislative measures. Data altruism is built upon the exercising of data subject’s rights, but of course, the fundamental right to data protection is not an absolute right – the law balances the rights and interests of individuals with respect to the use of their personal data, and the rights of others, including society (e.g., research). Further normative research, as well as a careful proportionality analysis of implicitly (or potentially explicitly, in the future) limiting data altruism in clinical research is thus necessary.

### 4.5. Remarks on the new intermediaries in the clinical research data value chain

Interviewees were concerned with the introduction of a new intermediary in clinical research – the data altruism organizations, with the role and responsibility of these organizations, and especially with how a trusted environment for the sharing of data (which is among the main aims of the DGA) will be created.

**Figure 1:**
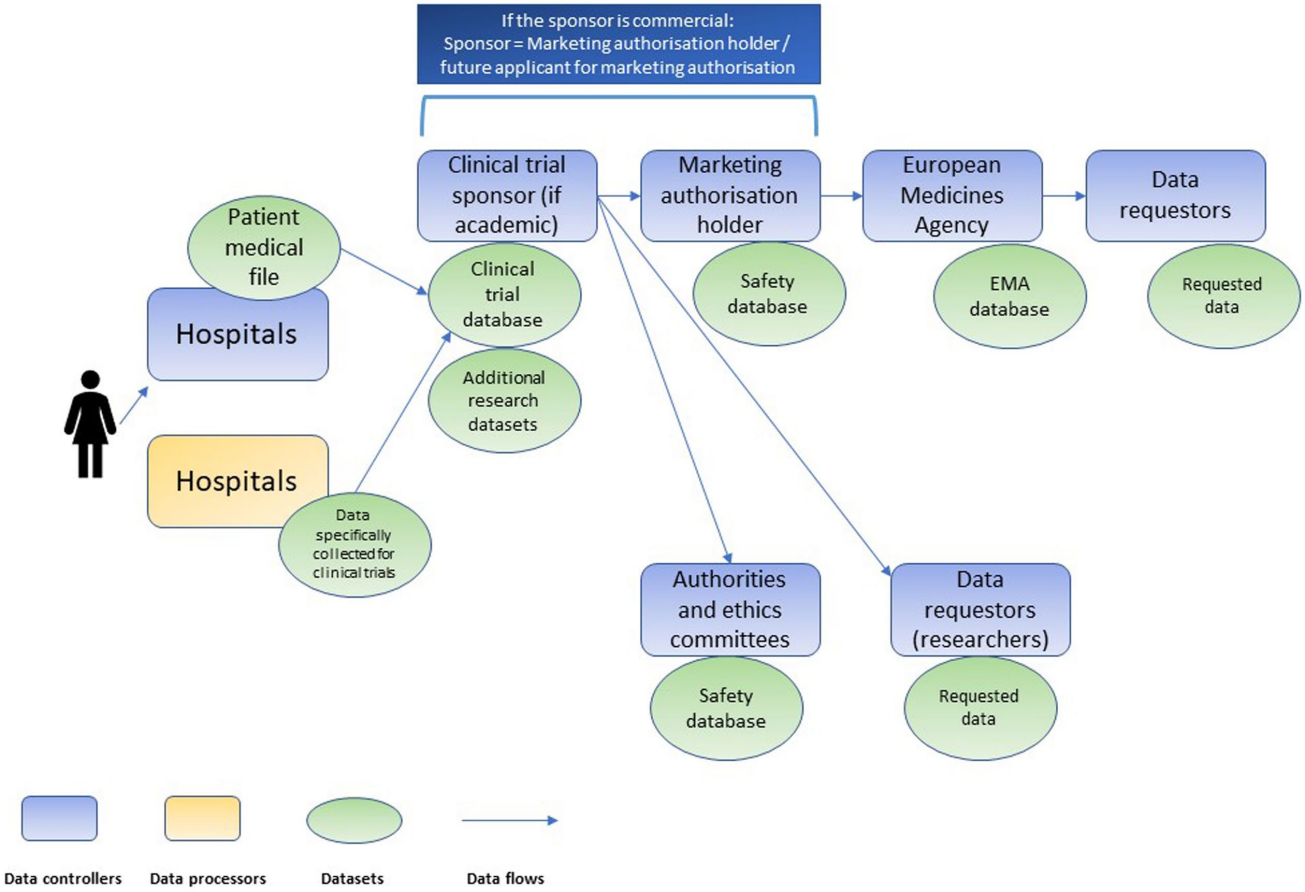
Chain of independent data controllers in clinical trials, adapted from Negrouk et al. (26).

**Figure 2:**
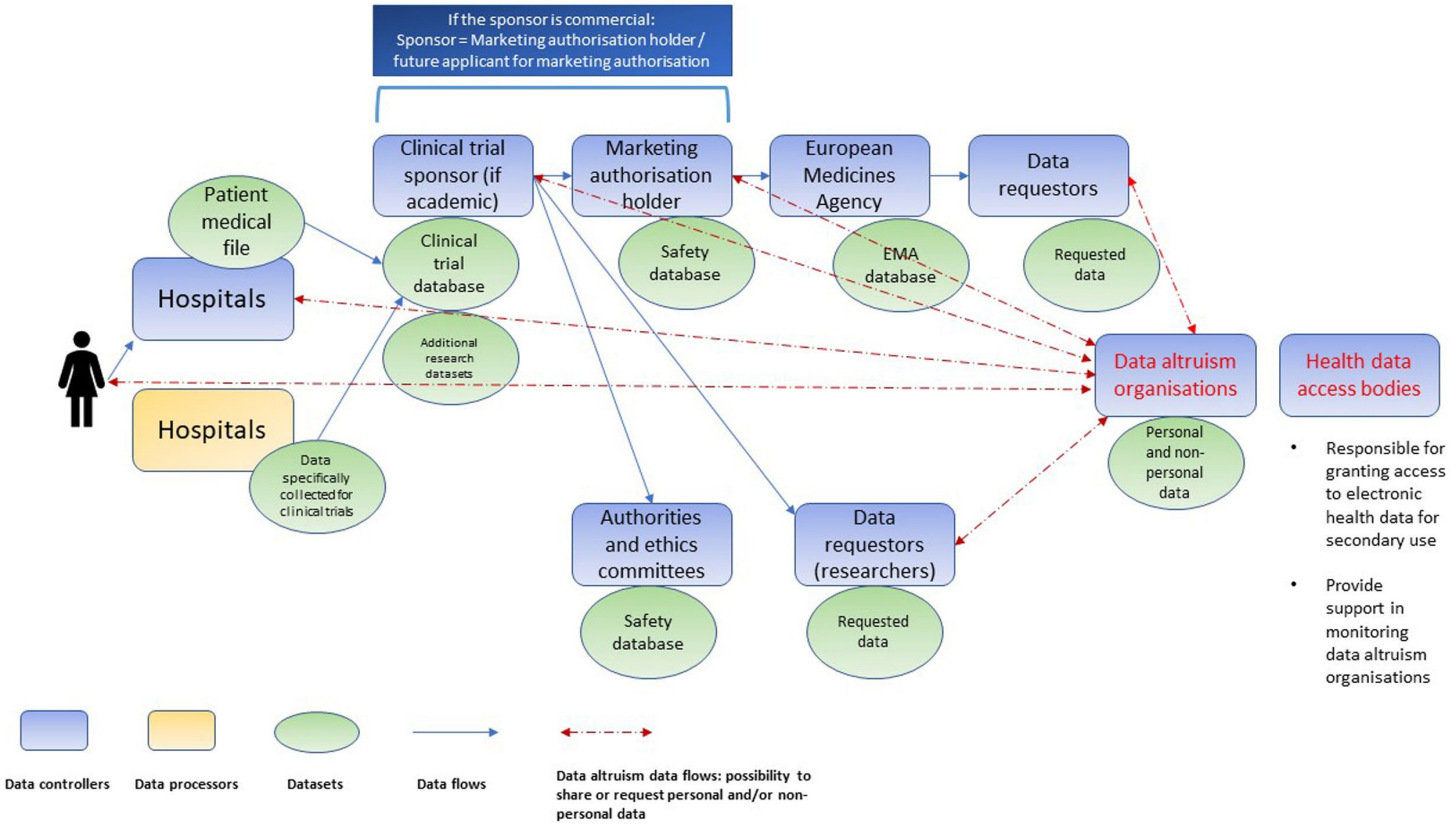
Chain of independent data controllers in clinical trials after the introduction of data altruism organizations and health data access bodies (non-exhaustive illustration).

Clinical research is characterized by a complex data value chain. Various controllers (e.g., hospitals, clinical trial sponsors, market authorization holders, biobanks, registries, regulatory agencies and others) conduct processing activities on the same personal data but following their own respective purposes (see Figure 1). Both the DGA, and, since the conduct of the interviews, the EHDS proposal introduce new intermediaries in the chain: data altruism organizations and health data access bodies, respectively (Figure 2). Data altruism organizations should be legal persons that “seek to support objectives of general interest by making available relevant data based on data altruism at scale.”^31^ They should be able to collect relevant data from natural and legal persons or to process data collected by others, acting as controllers or processors.^32^ In order to register as and use the label “data altruism organization recognized in the Union”, they should meet the requirements laid down in the DGA, namely: (1) operate on a not-for-profit basis and be legally independent from any entity that operates on a for-profit basis; (2) carry out data altruism activities through a structure that is functionally separate from its other activities, and (3) comply with the rulebook referred to in Article 22(1) DGA.^33^

Health data access bodies (to be designated by each EU Member State) will be public service bodies, responsible for granting access to electronic health data for secondary use.^34^ Their tasks include, *inter alia*, contributing to data altruism activities, more specifically by supporting the competent authorities (established under Article 23 DGA) in the monitoring of data altruism organizations.^35^

As discussed above, one of the general aims of both the DGA and EHDS is to increase trust in the data value chain and establish control for individuals over their personal data. However, at present, neither the DGA, nor the EHDS proposal, in our view, appear to provide sufficient reasons to inspire trust, as discussed throughout this section and further explained below. The findings are also in line with the high-level conclusions of a recent report about the results of EU-wide multistakeholder workshops focused on data altruism for the future EHDS (41).

Below, we briefly discuss relevant questions pertaining to each of the two new bodies.

#### 4.5.1. Data altruism organizations

As regards the role of data altruism organizations, the main question to put forward is whether data subjects can trust that their data will be processed only under the conditions and for the purposes that they consented upon or gave permission for (11). The DGA proposal aimed to provide an answer in the then-Article 19, in which it promised to establish a sort of a “policing obligation” for data altruism organizations, namely, to ensure that the altruistically shared data is not to be used by data users for other purposes than those of general interest for which the organization permits the processing. Data altruism organizations were originally foreseen as “supervisors and enforcers” which would verify and police the relevant safeguards (i.e., consent, revocability, purpose restriction) (42). Although this policing obligation was riddled with uncertainties (11) it was nevertheless an attempt to bring trust in the value chain. However, it does not exist in the final text of the adopted DGA. It is replaced with a requirement geared toward data altruism organizations themselves to “not use the data for other objectives than those of general interest,”^36^ which could be understood to mean that data altruism organizations shall not make the data available for other objectives than general interest ones, but that they are exempt from controlling whether data reusers process the data lawfully.

The omitting of citizen representatives, and in particular patient representatives from the decision-making process within data altruism organizations could also be viewed as a weakness of the system, especially considering again the DGA goal to enhance trust into data sharing. Interviewees also commented on the need for more safeguards for data altruism organizations.

Oversight mechanisms, such as ethical oversight, are one of the possible ways to establish trust. Recital 46 of the DGA does specify that safeguards for data altruism “should include (…) *oversight mechanisms such as ethics councils or boards, including representatives from civil society* to ensure that the data controller maintains high standards of scientific ethics and protection of fundamental rights.” [authors’ emphasis] However, with respect to health research, it is not clear whether the ethics councils are supposed to be the existing research ethics committees, or rather some kind of in-house ethics boards, part of data altruism organizations (11). Moreover, although clinical trials are subject to obligatory ethical review, this is not the case for all other types of medical research, as the matter is regulated in a divergent way across different EU countries. For instance, in Sweden, research that involves special categories of personal data needs to be reviewed by an ethics committee, whereas this is not the case for Latvia (43). Therefore, data subjects who engage in altruistic sharing of their personal data but reside in different EU Member States, would not be benefitting equally from ethics review as an oversight mechanism.

Bearing in mind the foregoing, establishing a role for civil society representatives within data altruism organizations becomes even more important, as this oversight mechanism would not depend on divergent national rules for ethical review. Health research is increasingly influenced by a Europe-wide trend toward a patient-focused approach (44). The concept of patient empowerment, as well as the related notions of patient involvement and engagement hold center stage in this trend (45). The aim is to ensure that patients’ needs and priorities in healthcare and research are identified and met. Patient empowerment occurs at all stages of the research and development cycle, e.g., design, conduct, or communication. It takes different forms, for instance patient advisory panels in pharmaceutical companies or research institutes, or patient representatives in the working groups of regulatory bodies such as the European Medicines Agency (EMA) (46). A patient advisory panel would be a welcome addition to data altruism organizations that specialize particularly in collecting and making available of data for health research purposes (it could easily be envisaged that data altruism organizations would specialize per sector, due to the complexity of managing diverse types of data for diverse fields and purposes). However, without a provision in the DGA articles that corresponds to Recital 46, it remains uncertain whether data altruism organizations would have sufficient incentives to work with civil society representatives. In the case of health research, an ample opportunity to establish patient involvement in data altruism organizations could be the EHDS proposal, but the legislator did not opt for this in the first draft published by the European Commission.

Finally, data altruism organizations, in carrying out data altruism activities, would act as data controllers pursuant to GDPR.^37^ As such, they would be engaging in controller-to-controller transfers of personal data. Independent controller-to-controller transfers, although implicit in the GDPR,^38^ are not specifically named in the regulation. There have long been calls from clinical research stakeholders for clarification of the relationship between independent controllers, particularly the ones that form the complex clinical research data value chain (24). The EHDS, as the sector-specific piece of legislation that aims to establish mechanisms for data altruism in the health sector (14), should ideally provide clarifications on this subject. A national example that could be a useful inspiration in this regard can be found in the Belgian implementation of the GDPR. In particular, in the context of further processing of personal data and the implementation of Article 89 GDPR, the law mandates that the original controller concludes an agreement with the new controller. This agreement should contain the contact details of both controllers, and the reasons why the exercise of data subjects’ rights is likely to make the achievement of the further processing impossible or seriously hinder it.^39^ According to scholars, the underlying assumption of the law is that the original controller would thereby act as a contact point for the data subjects (47).

#### 4.5.2. Health data access bodies

Several remarks can be put forward as regards the health data access bodies established with the EHDS proposal. The proposal shows a positive evolution in the direction of stakeholder involvement, as it mandates that health data access bodies shall “actively cooperate with stakeholders’ representatives, especially *with representatives of patients*” [authors’ emphasis].^40^ Although this obligation lacks further specification in concrete terms in the first draft of the EHDS proposal (as published by the European Commission), and thus risks being turned into an empty promise, the recent Draft report on the EHDS proposal, prepared by the European Parliament’s Committee on the Environment, Public Health and Food Safety (ENVI) and Committee on Civil Liberties, Justice and Home Affairs (LIBE) takes a further step toward strengthened patient involvement (48). Specifically, the ENVI and LIBE committees proposed that the relevant article be amended as follows: “Member States shall ensure that essential health stakeholders’ representatives, including patient organizations and healthcare professional shall be present in the governance and decision-making structures of the health data access bodies (…).” It remains to be seen how the draft legislation will evolve in the future.

Additionally, it must be critically observed that health data access bodies, which are foreseen as intermediaries/service providers,^41^ will be involved in the monitoring of the activities of another intermediary/service provider (data altruism organizations). Questions can be put forward as regards the potential conflict of interest. Related to this, the EDPB and EDPS already criticized the applicable provision in the EHDS proposal (Article 40) for being “unclear, particularly with regards to the interplay with the respective provision introduced by the DGA” and called for its clarification (49).

## 5. Future research

Data altruism in clinical research will rely on patients’ willingness to engage in altruistic data sharing. Therefore, in addition to gathering insights on the opinions of sponsors, investigators and ethics committees, as done in this paper, it is key that the voice of the patient community is included in future research endeavors on this topic, to better understand how the mechanism could be best suited to patients’ needs. More specifically, surveys, semi-structured interviews, focus groups or workshops could be employed to collect the patient perspective. A positive example in this regard are the three-day EU-wide multi-stakeholder workshops organized by the European Commission-funded project “Toward European Health Data Space” (TEHDAS), at which civil society representatives comprised 23% of all participants (41). Furthermore, it is not clear at present whether the EU legislator aims at the application of data altruism in the clinical research field. Further normative research, as well as a careful proportionality analysis on the need (or not) to limit the full operationalization of data altruism in clinical research is necessary. Finally, careful investigation – through use cases – of the organizational aspects relating to the roles of data altruism organizations, health data access bodies, and other competent bodies (such as data protection authorities and research ethics committees) would be valuable.

## 6. Strengths and limitations

To the best of our knowledge, this is the first scholarly contribution on the topic of data altruism to contain a qualitative research component. Several limitations could be listed for this study. Although qualitative evidence cannot be generalized by nature, the insights of the participating experts could be perceived as a meaningful indication of the early perceptions of data altruism. The interviews were conducted based on the DGA proposal. It would thus be valuable to explore in the future how perspectives on the data altruism mechanism, as introduced in the final text of the DGA, have changed. In addition, related to the question of reflexivity in qualitative research, it was acknowledged that all researchers involved in the study have professional experience in the legal framework for clinical trials and data protection, and some have an interest in patient empowerment and patient engagement, which could have influenced the semi-structured interviews design and conduct, as well as the legal analysis. Data altruism is a mechanism aimed to be applied irrespective of the sector, hence the discussion presented here in relation to clinical research might not be directly applicable in the context of other fields. Due to time and logistical constrains, a single researcher (TL-S) performed all interviews and conducted the full analysis of the interview data. Although no cross-check was performed, the use of the other stages of the framework method, and the availability of existing literature to inform the coding process, minimized subjective interpretation of the data. With respect to legal analysis, an important limitation is the discussion on the EHDS, as the proposed regulation is not final yet and is currently undergoing tough revisions.

## 7. Conclusion

The article elucidated challenges and uncertainties pertaining to the data altruism mechanism and its application in clinical research through a combination of empirical research on the views of clinical research stakeholders and legal doctrinal research. The data altruism mechanism introduced with the DGA holds the potential to facilitate data sharing, and to further foster and harmonize data altruism practices across the EU. However, at the moment, the application of the concept in practice is still riddled with uncertainties and challenges, particularly in the field of clinical research. The interplay of the DGA rules with the provisions of the GDPR and the EHDS proposal is also insufficiently clear and further efforts from the legislator are required to build a working, patient-centered, and research-fostering data altruism system.

## Data availability statement

The datasets presented in this article are not readily available because of legal and ethical constraints. Study participants did not provide consent for the sharing of interview transcripts with parties other than the researchers. The interview transcripts cannot be made available, to make sure that the confidentiality and privacy of the interviewees is preserved. Requests to access the datasets should be directed to teodora.lalova@kuleuven.be.

## Ethics statement

The studies involving human participants were reviewed and approved by Ethics Committee Research UZ/KU Leuven, file number S65106. The participants provided their written informed consent to participate in this study.

## Author contributions

TL-S designed the study. TL-S, IH, and JM further discussed the design of the study. TL-S collected and analyzed the data and drafted the manuscript. JM and IH thoroughly reviewed the manuscript. All authors approved the final version of this manuscript.

## Funding

TL-S’s Ph.D. at KU Leuven was supported with a scholarship awarded by the Research Foundation–Flanders (FWO), Project No. 11H3720N. This study was supported by IMI CARE. The CARE project has received funding from the Innovative Medicines Initiative 2 Joint Undertaking (JU) under grant agreement No 101005077. The JU receives support from the European Union’s Horizon 2020 research and innovation programme and EFPIA and BILL & MELINDA GATES FOUNDATION, GLOBAL HEALTH DRUG DISCOVERY INSTITUTE, UNIVERSITY OF DUNDEE. The content of this publication only reflects the authors’ view and the JU is not responsible for any use that may be made of the information it contains.

## Conflict of interest

The authors declare that the research was conducted in the absence of any commercial or financial relationships that could be construed as a potential conflict of interest.

## Publisher’s note

All claims expressed in this article are solely those of the authors and do not necessarily represent those of their affiliated organizations, or those of the publisher, the editors and the reviewers. Any product that may be evaluated in this article, or claim that may be made by its manufacturer, is not guaranteed or endorsed by the publisher.

## Footnotes

^1^Article 1, Recital 3 and 5 DGA.

^2^Article 2(8) DGA.

^3^Article 2(16) DGA.

^4^Chapter IV DGA.

^5^Recital 50 DGA.

^6^Article 2(9) DGA.

^7^The Health Data Hub (HDH) is an infrastructure created in November 2019, that is intended to facilitate the sharing of health data from a wide variety of sources in order to promote research. HDH may refer to (1) the public interest group which is responsible for setting up and administering the platform, and (2) the technical solution, namely a technological platform allowing the storage and provision of data. More information available at: CNIL. La Plateforme des données de santé (Health Data Hub), 9 February 2021, https://www.cnil.fr/fr/la-plateforme-des-donnees-de-sante-health-data-hub.

^8^Article 25 DGA.

^9^Recital 50 DGA.

^10^Recital 36, 50, Chapter IV DGA.

^11^Recital 50 DGA: "In accordance with Regulation (EU) 2016/679, scientific research purposes could be supported by consent to certain areas of scientific research where in keeping with recognized ethical standards for scientific research or only to certain areas of research or parts of research projects."

^12^Broad consent was very restricted by the interpretation of the EDPB in its Guidelines 05/2020 on consent under Regulation 2016/679. See also reference (11).

^13^Article 6(1)(a) GDPR.

^14^Article 6(1)(f) GDPR.

^15^Article 6(1)(e) GDPR.

^16^See, e.g., (14) Explanatory memorandum to the proposal on the EHDS, p. 13: "On secondary use of electronic health data, researchers, innovators, policy makers and regulators would be able to have access to quality data for their work in a secure way, with a trusted governance and at lower costs *than relying on consent*." [authors’ emphasis]; Recital 37 EHDS proposal: "(…) *This Regulation provides the legal basis in accordance with Articles 9(2) (g), (h), (i) and (j) of Regulation (EU) 2016/679* for the secondary use of health data, establishing the safeguards for processing, in terms of lawful purposes, trusted governance for providing access to health data (through health data access bodies) and processing in a secure environment, as well as modalities for data processing, set out in the data permit. (…)" [authors’ emphasis].

^17^ https://www.merriam-webster.com/dictionary/typically

^18^Idem.

^19^Such a strict interpretation would follow based on the Article 29 Working Party view in its "Opinion on purpose limitation" (not endorsed by the EDPB). The WP29 viewed the very first processing activity (i.e., the collection) as separate from all subsequent processing operations, and as such only the collection of personal data qualified as the initial (primary) processing, while all subsequent activities were considered "further processing." See Article 29 Data Protection, Working Party, Opinion 03/2013 on purpose limitation, 00569/13/EN, WP 203, 2 April 2013, (‘Article 29 WP, WP203’).

^20^Article 5(1) and Article 6(4) GDPR.

^21^See Recital 50 GDPR: "(…) The processing of personal data for purposes other than those for which the personal data were initially collected should be allowed only where the processing is compatible with the purposes for which the personal data were initially collected. In such a case, no legal basis separate from that which allowed the collection of the personal data is required (…)."

^22^Article 2(8) DGA.

^23^Article 2(16) DGA.

^24^Recital 30 DGA.

^25^Article 20 GDPR.

^26^Recitals 5, 12 EHDS proposal.

^27^Article 4(1), Recital 31 DA proposal.

^28^Article 3(8) EHDS proposal: "Natural persons shall have the right to give access to *or request a data holder from the health or social security sector to transmit their electronic health data to a data recipient of their choice from the health or social security sector*, immediately, free of charge and without hindrance from the data holder or from the manufacturers of the systems used by that holder." [authors’ emphasis].

^29^Article 15 GDPR.

^30^Article 15(1) and (3) GDPR.

^31^Recital 3 DGA.

^32^Recital 50 DGA.

^33^Article 18 DGA.

^34^Article 36 EHDS proposal.

^35^Article 37(h), Article 40 EHDS proposal.

^36^Article 21(2) DGA.

^37^Article 4(7) GDPR.

^38^Article 14 GDPR.

^39^Articles 194–195 of the Law of 30 July 2018 on the protection of natural persons with regard to the processing of personal data/Loi relative à la protection des personnes physiques à l’égard des traitements de données à caractère personnel 30 Juillet 2018.

^40^Article 36(3) EHDS proposal.

^41^As their tasks include deciding on data access applications, authorizing and issuing data permits to access electronic health data, etc. (Article 37(1)(a) EHDS proposal).
