# Supplementary material for: The application of data altruism in clinical research through empirical and legal analysis lenses
Source: Front Med (Lausanne). 2023 Mar 30;10:1141685. doi: 10.3389/fmed.2023.1141685 (PMC10098212; doi:10.3389/fmed.2023.1141685)
Supplement: Supplementary file 1 [file Data_Sheet_1.pdf]

Supplementary Material 1

Interview guide (excerpt with the question dedicated to data altruism)

- **Present yourself (name and affiliation)**
- **Thank the interviewee** for their participation in the project.
- Explain **very briefly the purpose** of the interview, state its duration (apprx 1 h), and where the results will be implemented.
  - *This interview is the follow-up to an online survey. Both the interview and the survey are part of a study with which we want to gather evidence about the experience of relevant stakeholders involved in clinical research on key challenges and related possible solutions concerning the compliance with data protection rules prior to and during the COVID-19 pandemic.*
  - *The results will **be incorporated** in scientific publications, PhD dissertation, and will also serve to guide the IMI CARE consortium in being GDPR compliant.*
- Put the interviewee **at ease**:
  - *No wrong answers*
  - *Digitally recorded*
  - *Confidential, anonymous*
  - *Voluntary, do not have to answer anything they do not feel comfortable answering*
  - *They can stop the interview at any time, without having to give a reason*
- Ask the interviewee if they have any **questions** before the start of the interview.
- **Explain how the interview will proceed**, e.g.*:*

We will start with some warming-up questions. Subsequently, we will focus on the research questions, using as a basis the online survey in which you have participated. We will look into primary and secondary use of personal data for clinical studies, transparency, data control, communication with ethics committees, and challenges encountered prior to and during the pandemic.

- **Ask for permission to turn on the recording function.**

Warming-up questions:

- Can you tell me a little bit about yourself and your background?
- To what extent do you have experience with clinical research?

(...)

Data altruism (same question for all stakeholder groups):

There is a new legislation on data sharing at the European level (the Data Governance Act), wherein the concept 'data altruism' is proposed. This refers, among others, to 'the consent by data subjects to process their personal data, for purposes of general interest, such as scientific research purposes or improving public services'. What is your view of the newly proposed data altruism mechanism?
